# Lateral Epicondylitis of the Elbow in an Airline Pilot Regarding Throttle Lever Operation

**DOI:** 10.7759/cureus.49634

**Published:** 2023-11-29

**Authors:** Naoya Sawamoto

**Affiliations:** 1 Department of Aerospace Medicine, Sapporo Ginnankai Memorial Hospital, Sapporo, JPN

**Keywords:** presenteeism, aviation medicine, occupational health, aerospace medicine, pilot, aviator, lateral epicondylitis, tennis elbow

## Abstract

Lateral epicondylitis is a degenerative condition affecting the origin of the extensor tendon in the forearm. It is the primary etiology of elbow pain in adults and is also a frequently reported ailment in various occupational sectors. Herein, we present a case of lateral epicondylitis in an airline pilot. The etiology was supposed to be the frequent and prolonged use of throttle levers in the at-risk limb position, i.e., forearm pronation and wrist dorsiflexion. Despite limited treatment options due to aeromedical standards, the patient recovered after approximately one month, primarily by means of physical therapy and improving the working posture. In general, pilots are subject to strict medical standards and may not receive standard treatments. There are also reports of treatment withdrawal and concealment of disease due to fear of losing their license. The treatment of pilots requires special consideration based on doctor-patient trust and occasionally collaboration with experts in aviation medicine, such as aeromedical examiners.

## Introduction

Lateral epicondylitis (LE) of the elbow, also known as tennis elbow or lateral elbow tendinopathy, is a degenerative condition affecting the origin of the extensor carpi radialis brevis (ECRB) [1]. It predominantly affects individuals aged 30-50 years, causing pain from the lateral elbow to the forearm during activities such as gripping and lifting objects or squeezing towels, leading to impairments in daily activities [2]. LE diagnosis is typically based solely on clinical history and physical tests such as Mill’s test, Cozen’s test, or Maudsley's test [3]. Although the prevalence varies around 1%-5% depending on reports in the general population, it is recognized as the most common cause of elbow pain in adults [2,4].

A variety of studies have examined LE in the context of occupations, underscoring its significance as an occupational health issue [1]. Work-related risk behaviors include repetitive hand or forearm movements and use of heavy tools, with a higher prevalence noted in industries such as manufacturing, construction, and food processing [1,4]. Despite a reported 90% remission rate, other studies found a high recurrence rate or more than half of the cases with residual symptoms [2,5]. Given that work-related LE is particularly resistant to treatment, LE can become a chronic condition in workers resulting in frequent and prolonged absences and a potential loss of productivity [1,2].

We present a case of LE in an airline pilot associated with throttle lever manipulation. To date, the literature reporting LE in pilots is sparse, and the occupational risks may have been under-recognized [6]. Pilots may face work restrictions due to strict medical standards, which may lead them to conceal their illness or interrupt treatment to avoid flight suspension [7]. Special considerations are therefore required when providing health care to pilots. This report outlines the approach to the disease from an aeromedical perspective and identifies methods for explaining and administering treatment to pilots. This information will also be useful for general practitioners managing other afflictions in pilots.

## Case presentation

The patient was a 60-year-old, right-handed, Japanese airline pilot (captain) with no history of musculoskeletal issues including trauma. There was no history of LE or elbow pain suggestive of LE. Further, the patient had not undertaken any sport recently. He had more than 30 years of commercial jet experience, mainly on long-haul routes. Approximately two months after starting work on short-haul turboprop aircraft, which was a new type of plane for him to fly, the patient began to experience mild pain in the lateral part of the right elbow during movement.

Physical examination revealed mild tenderness over the lateral epicondyle of the right elbow, but no radiating pain or Tinel's sign. There were no inflammatory findings such as erythema, swelling, or localized heat, and no deformity was observed in the right elbow. The joint range of motion was normal. No clinical findings were observed in the other parts of the body. In contrast, physical tests for LE, including Mill's test, Cozen's test, and Maudsley's test, were all positive. Given the patient’s age, history, and these clinical findings, LE was strongly suspected, as the differential diagnoses of radial tunnel syndrome, snapping elbow, osteochondritis dissecans, and rheumatoid arthritis were unlikely.

**Figure 1:**
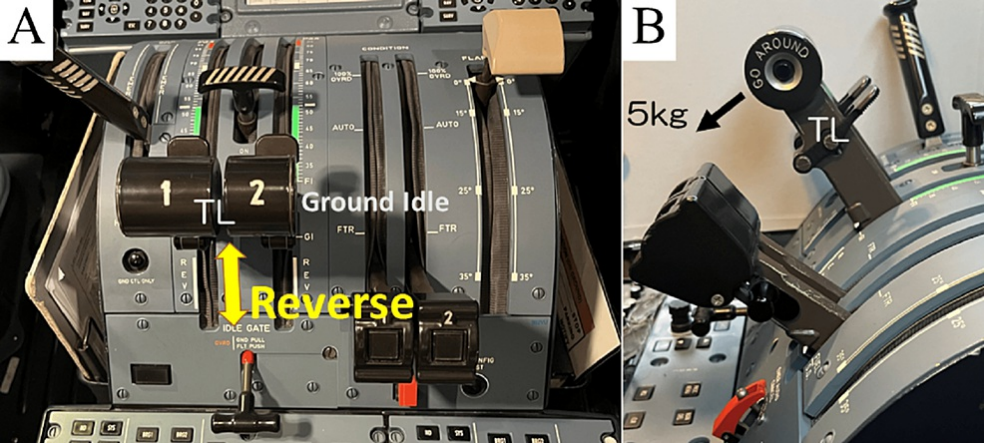
Throttle lever configuration (A) Two throttle levers are attached to the left side of the center pedestal, and the patient sitting in the left seat operates the levers simultaneously with their right hand. Full reverse is 45 degrees and neutral position (ground idle) is 60 degrees when the horizontal line to the ground toward the rear of the aircraft is 0 degrees. (B) Each lever requires about 2.5 kg, i.e., a total of 5 kg of force, to move according to the measurements. TL: throttle levers

**Figure 2:**
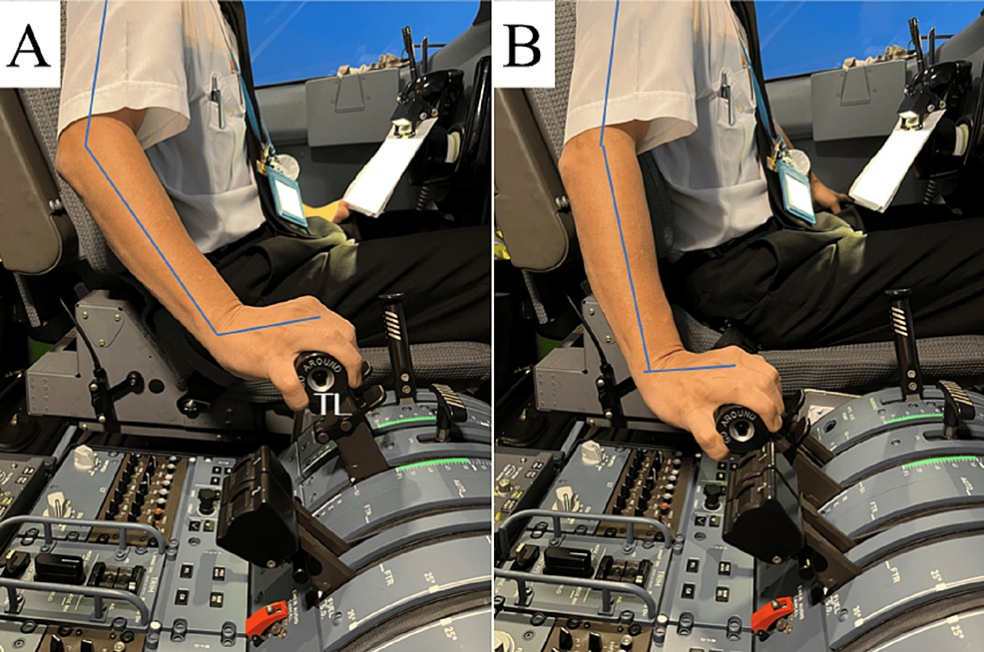
Throttle operation (A) Neutral position (ground idle) and (B) maximum reverse position. The more the elbow joint was extended, the greater the wrist dorsiflexion to pull the throttle. The figures used in the article do not depict the patient. TL: throttle levers, Blue line: axis of limbs

The medical interview revealed no evidence of risky movement behavior in daily life. However, the patient mentioned that since beginning work on the new airplane model, there had been more opportunities to apply prolonged force to the throttle levers. The throttle is operated by the pilot in the cockpit and controls fuel flow. Moving forward increases power and moving backward decreases power. The rearmost part has a reverse position that produces a reverse thrust to slow down on the ground. In this detent, the more it is moved backward, the stronger the reverse thrust becomes. A detailed study of the working environment revealed that the throttles must be in the reverse position almost constantly during ground movement, that is taxiing (Figure 1A). The reverse detent of the throttle has a spring to return to the neutral position (ground idle), and according to our measurements, requires approximately 5 kg of pressure (Figure 1B). This maneuver was accomplished by grasping the power levers with the pronated forearm, dorsiflexing the wrist joint, and extending the elbow joint to pull the power lever. The greater the extension of the elbow joint, the greater the wrist dorsiflexion (Figures 2A, 2B). The throttles tend to be gripped tightly to withstand the heavy movement force and fulfill the need for precise adjustments. He was involved in short-haul routes, which consisted of several round-trip flights per day with a flight time of approximately 30 to 40 minutes. The average taxi time was seven minutes, and this process was repeated four to six times a day. The patient's throttle manipulation, including its frequency, is consistent with reported risks for LE. Based on the physical findings and his history, we concluded that his diagnosis was LE.

**Figure 3:**
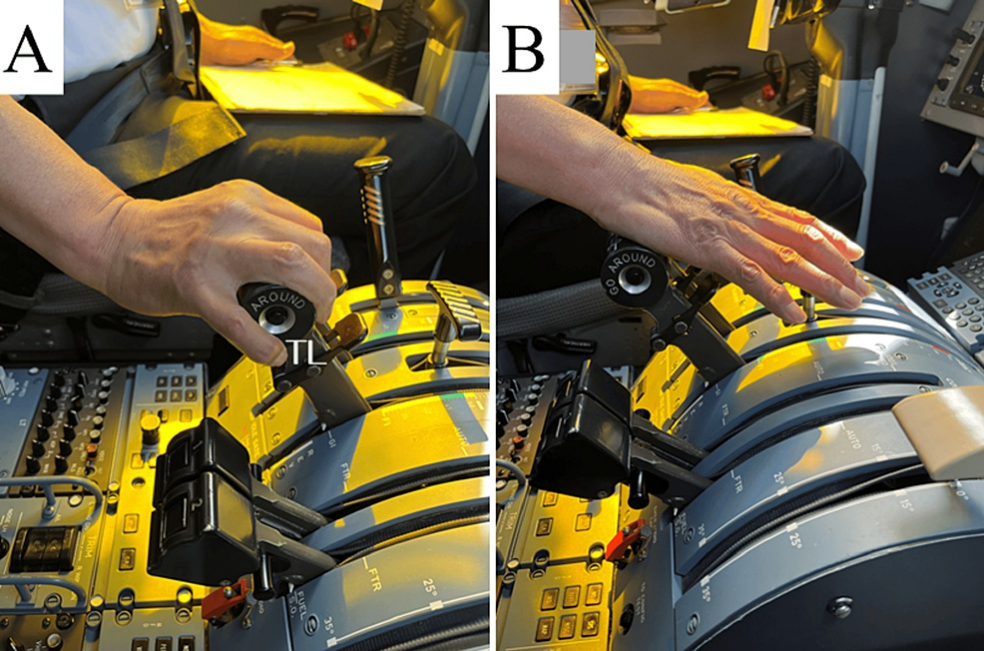
Improvement of the operation The throttle lever was operated by gripping (A) but was changed to pushing with the base of the palmaris (B), avoiding dorsiflexion of the wrist joint. The figures used in the article do not depict the patient. TL: throttle levers

Careful questioning revealed no loss of grip, other muscular strength, decreased range of motion, and mild pain. Thus, we confirmed that there would be no disruptions to the airline operations. Treatment options include oral nonsteroidal anti-inflammatory drugs or steroid injections, both of which were deemed inappropriate for use by pilots under Japanese regulation. After an explanation of the treatment options and prognosis, including their impact on the patient’s flight duties, the patient opted to continue working. Therefore, we prescribed physiotherapy and discussed ways to avoid high-risk movements without compromising safety. For physical therapy, the author, an orthopedic surgeon, instructed self-stretching of the extensor tendons at home twice a day according to the published protocol [8]. We also advised the patient to manipulate the throttle at the base of the palm, similar to some other pilots at the company, to avoid dorsiflexion of the wrist (Figures 3A, 3B). The patient complied with this instruction and physiotherapy and continued working as a pilot. The symptoms gradually improved and the pain disappeared after approximately one month. After six months, the patient continued to work without any problems.

## Discussion

This is a case of LE in an airline pilot related to throttle lever operation. The cause of LE was assumed to be the continuous dorsiflexion of the wrist joint with the forearm pronated, which exerted an undue force on the attachment of the ECRB tendon.

LE is considered an enthesopathy of ECRB tendon attachment, and several reports indicate a significant association with occupational activities [1]. These activities include forearm pronation, utilization of tools weighing over 1 kg, forceful hand gripping, and repetitive wrist joint movements [1,4]. In the present case, the patient was tightly gripping the throttle levers with a pronated forearm and dorsiflexed wrist while applying a force of approximately 5 kg. The patient performed short-haul, high-frequency operations, which led to prolonged exposure to repetitive high-risk behaviors. Thus, in this case, the throttle operation was considered a major risk factor for LE. Similar cases may arise in other pilots for two reasons. First, the market offers a limited number of models of turboprop airliners, all with similar cockpit designs [9]. Second, turboprop aircraft are frequently employed on short-haul, high-frequency routes [9]. Overall, occupational factors should be carefully discussed during the treatment of LE.

The development of illnesses, including orthopedic diseases, is a significant concern for airline pilots regarding the continuity of their jobs [7]. Pilots are held to strict medical standards and failure to meet these criteria can result in disqualification from flying and, in severe cases, employment termination [7]. When assessing pilots, clinicians must keep in mind that even assigning an uncertain diagnosis may result in disruptions to the pilot’s employment. The following factors should be considered 1) the pilot's ability to continue flying with the medical condition; 2) treatment options and the applicability of standard treatments; and 3) the patient’s prognosis balancing return-to-work potential and the need for further testing. Throughout this process, it is imperative to ensure alignment with the medical standards set by aviation authorities as well as adherence to company regulations in the case of a professional pilot. This means that doctors may need to collaborate with an aeromedical examiner (AME) or an occupational doctor at the airline company. In the present case, the symptoms were mild and without any other symptoms, and the patient was assessed to be fit for airline operations under national regulations. Although some treatments were not recommended to accommodate the patient’s desire to continue working, we concluded that the symptoms could be ameliorated through conservative treatment and improvements in the workplace environment. Notably, pilots often have a strong inclination to continue flying, yet it is important to recognize that this may adversely affect their prognosis [7]. If the initial treatment did not alleviate the symptoms in the present case, more advanced interventions such as injections or oral treatment, along with a specified period of suspension from flying, would have been considered. Surgical intervention may be warranted in cases of LE refractory to other treatments. However, before planning irreversible treatments such as surgery, it is crucial to confirm that these treatments will not have a permanent impact on the patient’s medical certificate.

Pilots may not consistently report their illness, underscoring the need for special consideration in their medical care. Recent studies on commercial and military pilots suggest that pilots are likely not to report illnesses in order to continue flying or maintain their licenses [7]. Pilots have negative perceptions of medical visits and fear that disclosing their illness may negatively affect their work, leading them to withhold information from doctors [10]. This can lead to presenteeism among pilots, which ultimately has a detrimental effect on their long-term health due to the lack of adequate treatment [7]. In fact, in all documented cases of LE in pilots, they did not receive optimal treatment, and in some cases, treatment was dropped. Therefore, doctors must focus initially on building trust during interviews. To accomplish this, it is essential to convey a deep understanding of the patient’s profession and reassure them of our commitment to assist them in keeping them on board. In addition, seeking medical advice from specialists, such as an AME or occupational physician, is subject to patient consent and, if necessary, can be performed anonymously. Careful and professional treatment decisions must be made considering patients’ concerns and preferences.

## Conclusions

In the present case of LE in an airline pilot of a turboprop aircraft, the major cause was assumed to be the prolonged use of throttle levers with the forearm in a pronated position during taxiing. LE is a common occupational disease that underscores the importance of conducting thorough interviews regarding the relevant occupational factors. Given the strict medical standards for pilots, the diagnosis and treatment of their conditions should be carried out with careful attention to both national and company regulations while considering patient preferences. This necessitates a strong physician-patient relationship and sometimes collaboration with specialists, such as an AME, while maintaining privacy.

## References

[1] Bretschneider SF, Los FS, Eygendaal D, Kuijer PP, van der Molen HF (2022). Work-relatedness of lateral epicondylitis: systematic review including meta-analysis and GRADE work-relatedness of lateral epicondylitis. Am J Ind Med.

[2] Karabinov V, Georgiev GP (2022). Lateral epicondylitis: new trends and challenges in treatment. World J Orthop.

[3] Soares MM, Souza PC, Ribeiro AP (2023). Differences in clinical tests for assessing lateral epicondylitis elbow in adults concerning their physical activity level: test reliability, accuracy of ultrasound imaging, and relationship with energy expenditure. Int J Environ Res Public Health.

[4] van Rijn RM, Huisstede BM, Koes BW, Burdorf A (2009). Associations between work-related factors and specific disorders at the elbow: a systematic literature review. Rheumatology (Oxford).

[5] Vaquero-Picado A, Barco R, Antuña SA (2016). Lateral epicondylitis of the elbow. EFORT Open Rev.

[6] Farr RW (1982). Tennis elbow in aviators. Aviat Space Environ Med.

[7] Patel PK, Hoffman WR, Aden J, Acker JP (2023). Health care avoidance among Canadian pilots due to fear of medical certificate loss: a national cross-sectional survey study. J Occup Environ Med.

[8] Martinez-Silvestrini JA, Newcomer KL, Gay RE, Schaefer MP, Kortebein P, Arendt KW (2005). Chronic lateral epicondylitis: comparative effectiveness of a home exercise program including stretching alone versus stretching supplemented with eccentric or concentric strengthening. J Hand Ther.

[9] Guzhva VS, Curtis T, Borodulin V (2015). Market analysis for small and mid-size commercial turboprop aircraft. Int J Aviation Manag.

[10] Hoffman WR, Barbera RD, Aden J, Bezzant M, Uren A (2022). Healthcare related aversion and care seeking patterns of female aviators in the United States. Arch Environ Occup Health.

